# Decoding Hypercarotenemia: Integrating Pathophysiology, Clinical Recognition, and Precision Management

**DOI:** 10.7759/cureus.94519

**Published:** 2025-10-13

**Authors:** Zhilin Wang, Jiajun Xu, Guangming Xu, Yanguang Sha, Ke Wang, Guangbin Chen

**Affiliations:** 1 Graduate School, Wannan Medical College, Wuhu, CHN; 2 Department of Hepatobiliary Surgery, The Second People's Hospital of Wuhu, Wuhu Hospital Affiliated to East China Normal University, Wuhu, CHN; 3 Department of Medical Imaging, The Second People's Hospital of Wuhu, Wuhu Hospital Affiliated to East China Normal University, Wuhu, CHN

**Keywords:** carotenoid metabolism, genetic polymorphisms, hypercarotenaemia, metabolic disorders, personalized nutrition, β-carotene oxygenase

## Abstract

Hypercarotenemia represents a complex metabolic phenotype characterized by supraphysiological circulating carotenoid concentrations exceeding 300 μg/dL for β-carotene, manifesting as distinctive cutaneous xanthochromia with preserved scleral clarity - a critical differentiating feature from hepatobiliary dysfunction. The contemporary surge in detection rates, which has increased over the past decade, correlates with global dietary paradigm shifts, including the growth in plant-based diet adoption and the concurrent rise in metabolic dysfunction. This comprehensive review synthesizes current understanding of hypercarotenemia pathophysiology through systematic literature analysis encompassing molecular mechanisms, epidemiological trends, clinical phenotypes, and therapeutic interventions, with particular emphasis on genetic discoveries and precision management approaches.

Hypercarotenemia pathogenesis involves sophisticated interactions among three key mechanistic pathways: (1) intestinal absorption via SR-B1 receptors, (2) enzymatic conversion through β-carotene oxygenase 1 and 2 (Beta-carotene oxygenase 1 (BCO1)/BCO2) systems, and (3) genetic susceptibility primarily mediated by BCO1 variants (rs6564851, rs12934922, rs7501331). The condition demonstrates remarkable clinical heterogeneity influenced by individual metabolic capacity, intestinal microbiome composition, and concurrent endocrinopathies, particularly thyroid dysfunction and diabetes mellitus.

The management paradigms have evolved from simple dietary restriction to precision nutrition approaches, integrating genetic profiling with individualized tolerance thresholds while preserving established health benefits of carotenoid-rich diets. Standard diagnostic criteria incorporate both biochemical thresholds and functional assessments, including the retinol:β-carotene molar ratio as a functional measure of BCO1 activity. Although traditionally considered benign, hypercarotenemia serves as a valuable biomarker for underlying metabolic dysfunction and genetic variants affecting fat-soluble vitamin homeostasis, warranting clinical attention for risk stratification and personalized dietary counseling.

## Introduction and background

Carotenoids refer to a diverse family of over 600 naturally occurring pigments, primarily synthesized by plants, algae, and photosynthetic bacteria. These lipophilic compounds serve as the primary dietary sources of provitamin A, with β-carotene, α-carotene, and β-cryptoxanthin being the most significant contributors to human vitamin A status [1]. Common dietary sources include orange vegetables (carrots: 10-12 mg β-carotene/100g), green leafy vegetables (spinach: 8-10 mg/100g), and orange fruits (sweet potatoes: 6-8 mg/100g) [2].

The intersection of nutrition science and clinical medicine has revealed hypercarotenemia as a paradigmatic example of how dietary components interact with individual genetic and metabolic factors to produce clinically observable phenotypes. This condition, characterized by pathologically elevated circulating carotenoid concentrations exceeding 300 μg/dL for β-carotene (normal range: 50-300 μg/dL) [3], represents more than a cosmetic concern - it serves as a window into complex metabolic processes governing fat-soluble vitamin homeostasis. Hypercarotenemia should be understood as a metabolic phenotype rather than a discrete disease entity - representing the convergence of dietary exposure, genetic susceptibility, and metabolic dysfunction [4]. While often benign, it serves as a valuable biomarker for underlying metabolic processes and genetic variants affecting fat-soluble vitamin homeostasis, warranting clinical attention for risk stratification and personalized management.

The pathophysiological basis of hypercarotenemia centers on the unique biochemical properties of carotenoids. These lipophilic compounds demonstrate preferential accumulation in lipid-rich tissues, particularly the stratum corneum, while their hydrophobic nature prevents significant deposition in aqueous environments such as the sclera - a fundamental distinction from conjugated hyperbilirubinemia [5]. This tissue-specific distribution pattern produces the characteristic clinical presentation: prominent yellow-orange discoloration most evident in areas of thick keratinization (palms, soles, nasolabial folds) with conspicuous sparing of the sclerae [6].

Contemporary epidemiological data reveal a multifactorial increase in hypercarotenemia prevalence, driven by converging trends: the mainstream adoption of plant-based dietary patterns, widespread nutritional supplement utilization often exceeding recommended doses, and the rising burden of metabolic diseases that impair carotenoid processing [4,7]. Particularly concerning is the emergence of hypercarotenemia in specific vulnerable populations, including individuals with restrictive eating disorders, where altered adipose tissue dynamics fundamentally disrupts carotenoid storage and mobilization [8].

The clinical significance of hypercarotenemia extends beyond its visible manifestations. Recent molecular studies have uncovered associations with genetic variants affecting carotenoid-cleaving enzymes, suggesting that some cases may represent unrecognized inborn errors of metabolism [9]. Furthermore, the condition's frequent misdiagnosis as jaundice can lead to unnecessary investigations and patient anxiety, highlighting the need for improved clinical recognition and evidence-based management strategies [10]. This review provides a comprehensive synthesis of current knowledge regarding hypercarotenemia, integrating recent advances in molecular biology, clinical medicine, and nutritional science to present a unified framework for understanding this increasingly prevalent condition.

## Review

Molecular mechanisms underlying hypercarotenemia

Carotenoid Bioavailability and Absorption Dynamics

The journey of carotenoids from dietary sources to tissue deposition involves a sophisticated cascade of molecular events, each representing a potential regulatory node for hypercarotenemia development. Dietary carotenoids, predominantly obtained from plant matrices including β-carotene-rich vegetables (carrots, sweet potatoes), α-carotene sources (pumpkins, winter squash), and lycopene-containing fruits (tomatoes, watermelons), must first be released from their food matrix through mechanical and enzymatic digestion [11].

The bioavailability paradigm has evolved significantly with recognition that carotenoid absorption efficiency depends on multiple interacting factors. At the molecular level, carotenoid solubilization requires incorporation into mixed micelles-a process fundamentally dependent on concurrent dietary lipid intake [12]. This process requires bile salts (primarily cholate and deoxycholate) to emulsify carotenoids, followed by pancreatic lipase and cholesterol esterase action to facilitate micellar incorporation. Studies demonstrate that an optimal absorption rate was observed to occur with an intake of carotenoids with ≥5g dietary fat compared to fat-free meals [13].

The intestinal epithelium serves as a selective barrier, with enterocyte uptake mediated by scavenger receptor class B type 1 (SR-B1), whose expression levels show significant interindividual variation linked to genetic polymorphisms [14]. Recent investigations utilizing advanced metabolomics have revealed that the intestinal microbiome plays a previously underappreciated role in carotenoid metabolism [11].

Enzymatic Conversion and Metabolic Fate

The central dogma of carotenoid metabolism revolves around their enzymatic cleavage to generate retinoids-biologically active vitamin A derivatives essential for numerous physiological processes. Beta-carotene oxygenase 1 (BCO1) functions as a cytosolic enzyme essential for provitamin A carotenoid conversion, while BCO2 operates as a mitochondrial enzyme handling non-provitamin carotenoids (lycopene, lutein, zeaxanthin) [15]. BCO1, localized primarily in intestinal enterocytes and hepatocytes, catalyzes the symmetric cleavage of β-carotene at the central 15,15' double bond, yielding two molecules of retinal [16]. This reaction represents the rate-limiting step in vitamin A biosynthesis and serves as a critical control point preventing carotenoid accumulation under normal physiological conditions.

However, emerging evidence reveals substantial complexity beyond this simplified model. BCO2, initially characterized as a mitochondrial carotenoid-cleaving enzyme with broader substrate specificity, appears to function as a "metabolic gatekeeper," preventing excessive carotenoid accumulation through asymmetric cleavage reactions generating apocarotenoids [17]. The discovery of BCO2's role has profound implications for understanding hypercarotenemia, as genetic variants affecting its expression or activity could predispose individuals to carotenoid accumulation despite normal BCO1 function.

The tissue-specific expression patterns of these enzymes create metabolic compartmentalization, with implications for hypercarotenemia phenotypes. While intestinal BCO1 expression provides first-pass metabolism of absorbed carotenoids, hepatic enzyme activity determines systemic clearance rates. Importantly, extrahepatic tissues, including adipocytes, the kidney, and reproductive organs, express variable levels of both enzymes, contributing to the complex pharmacokinetics of carotenoid distribution and accumulation [18].

Genetic Determinants and Molecular Regulation

The genetic architecture underlying hypercarotenemia susceptibility has been progressively elucidated through genome-wide association studies (GWAS) and functional genomics approaches. The BCO1 gene harbors several functionally significant polymorphisms, with the rs6564851 variant emerging as a major determinant of conversion efficiency [19, 20]. Individuals homozygous for the variant allele demonstrate approximately 50% reduced enzyme activity, translating to significantly elevated circulating β-carotene levels following standardized dietary challenges [20].

Pharmacogenomic evidence indicates that rs6564851 carriers require a reduction in β-carotene supplement dosing to achieve equivalent serum retinol responses, while dietary interventions show enhanced efficacy in these individuals. Beyond single-nucleotide polymorphisms, emerging evidence implicates epigenetic regulation in carotenoid metabolism variability. The BCO1 promoter contains response elements for multiple transcription factors, including PPARγ, RXR, and ISX (intestine-specific homeobox), creating a complex regulatory network responsive to nutritional status, hormonal signals, and metabolic demands [21]. Particularly intriguing is the discovery that retinoic acid - the downstream product of carotenoid metabolism - induces ISX expression, which subsequently suppresses both BCO1 and SR-B1 transcription, establishing a negative feedback loop preventing excessive vitamin A accumulation [22].

The APOE genotype, traditionally associated with lipid metabolism and Alzheimer's disease risk, has emerged as an unexpected modulator of carotenoid homeostasis. APOE ε4 carriers demonstrate altered postprandial carotenoid responses and modified tissue distribution patterns, potentially explaining some of the unexplained variance in hypercarotenemia susceptibility [23].

Etiological factors and risk stratification

Dietary Patterns and Nutritional Epidemiology

Quantitative dietary assessment studies have established threshold effects, with daily β-carotene intake exceeding 20-30 mg consistently associated with clinically apparent hypercarotenemia within three months [3]. However, significant interindividual variability exists, with some individuals developing symptoms at lower intake levels while others remain asymptomatic despite higher consumption. This variability underscores the importance of host factors in modulating dietary responses. Safe reintroduction protocols recommend ≤7 mg/day for the general population [24], but the BCO1 variant carriers need more strict management.

Contemporary dietary pattern analysis reveals that hypercarotenemia risk correlates not merely with absolute carotenoid intake but with overall dietary quality indices and food matrix effects [25]. Individuals following strict plant-based diets, particularly raw food enthusiasts consuming large quantities of carrot juice or smoothies, represent a high-risk population due to both elevated intake and enhanced bioavailability from mechanical processing.

The role of dietary supplements deserves particular scrutiny given their concentrated nature and widespread use. Commercial β-carotene supplements, often marketed for their antioxidant properties or as "natural" tanning aids, can deliver pharmacological doses far exceeding dietary sources. Case series have documented hypercarotenemia following supplement regimens providing >30mg daily β-carotene-levels impossible to achieve through whole food consumption alone [26].

Metabolic Conditions and Endocrine Influences

The intricate relationship between systemic metabolism and carotenoid homeostasis manifests through multiple pathophysiological mechanisms. Thyroid dysfunction exemplifies this complexity: Mechanistically, thyroid hormone (T3) regulates retinoid X receptor (RXR) and peroxisome proliferator-activated receptor γ (PPARγ) expression [27], which in turn modulates BCO1 transcription through direct promoter binding.

The simplified pathway demonstrates: ↓T3 → ↓RXR/PPARγ → ↓BCO1 expression → ↑β-carotene accumulation, explaining the two-to-four fold elevation in serum carotenoids observed in untreated hypothyroidism. Clinical studies demonstrate that thyroid hormone replacement therapy can normalize elevated carotenoid levels, confirming the causal relationship [28].

Diabetes mellitus presents another metabolic state predisposing to hypercarotenemia through distinct mechanisms. Chronic hyperglycemia induces oxidative stress, potentially consuming antioxidant reserves and upregulating compensatory carotenoid absorption. Additionally, diabetic dyslipidemia alters the lipoprotein particles responsible for carotenoid transport, while concurrent hepatic steatosis impairs hepatic carotenoid metabolism [4]. The intersection of diabetes and hypercarotenemia may have prognostic implications, as some studies suggest elevated carotenoid levels in diabetics correlate with better glycemic control, raising questions about potential adaptive versus pathological responses [29].

Particularly intriguing is the hypercarotenemia observed in anorexia nervosa, where paradoxically elevated carotenoid levels occur despite restricted dietary intake. Recent metabolic studies reveal that severe caloric restriction triggers adipocyte lipolysis, releasing stored carotenoids into circulation. Simultaneously, reduced adipose tissue mass diminishes storage capacity, while hepatic dysfunction secondary to malnutrition impairs metabolic clearance [8]. This creates a "perfect storm" for hypercarotenemia development, with plasma levels inversely correlating with body mass index in affected individuals.

Genetic Susceptibility and Pharmacogenomic Considerations

The genetic landscape of hypercarotenemia susceptibility extends beyond simple Mendelian inheritance patterns to encompass complex gene-gene and gene-environment interactions. Large-scale genomic studies have identified multiple loci contributing to carotenoid metabolism efficiency, with cumulative genetic risk scores explaining interindividual variability in plasma carotenoid responses to standardized dietary interventions [30].

The BCO1 gene remains the most extensively characterized, with functional studies revealing that common variants affect not only enzyme activity but also substrate specificity and cellular localization. The rs12934922 polymorphism, for instance, alters enzyme stability at physiological temperatures, while rs7501331 affects post-translational modifications influencing enzymatic efficiency [31]. These variants often occur in linkage disequilibrium, creating haplotypes with distinct metabolic phenotypes.

Precision nutrition approaches link genetic variants to specific dietary recommendations. Emerging pharmacogenomic evidence suggests that genetic variants may also influence responses to therapeutic interventions. For example, individuals with reduced BCO1 activity show enhanced responses to dietary restriction but may require more aggressive management to achieve normal carotenoid levels [32]. This has led to proposals for genetic screening in cases of refractory hypercarotenemia or when considering high-dose carotenoid supplementation for therapeutic purposes.

Clinical manifestations and diagnostic approaches

Dermatological Presentations and Differential Diagnosis

**Figure 1:**
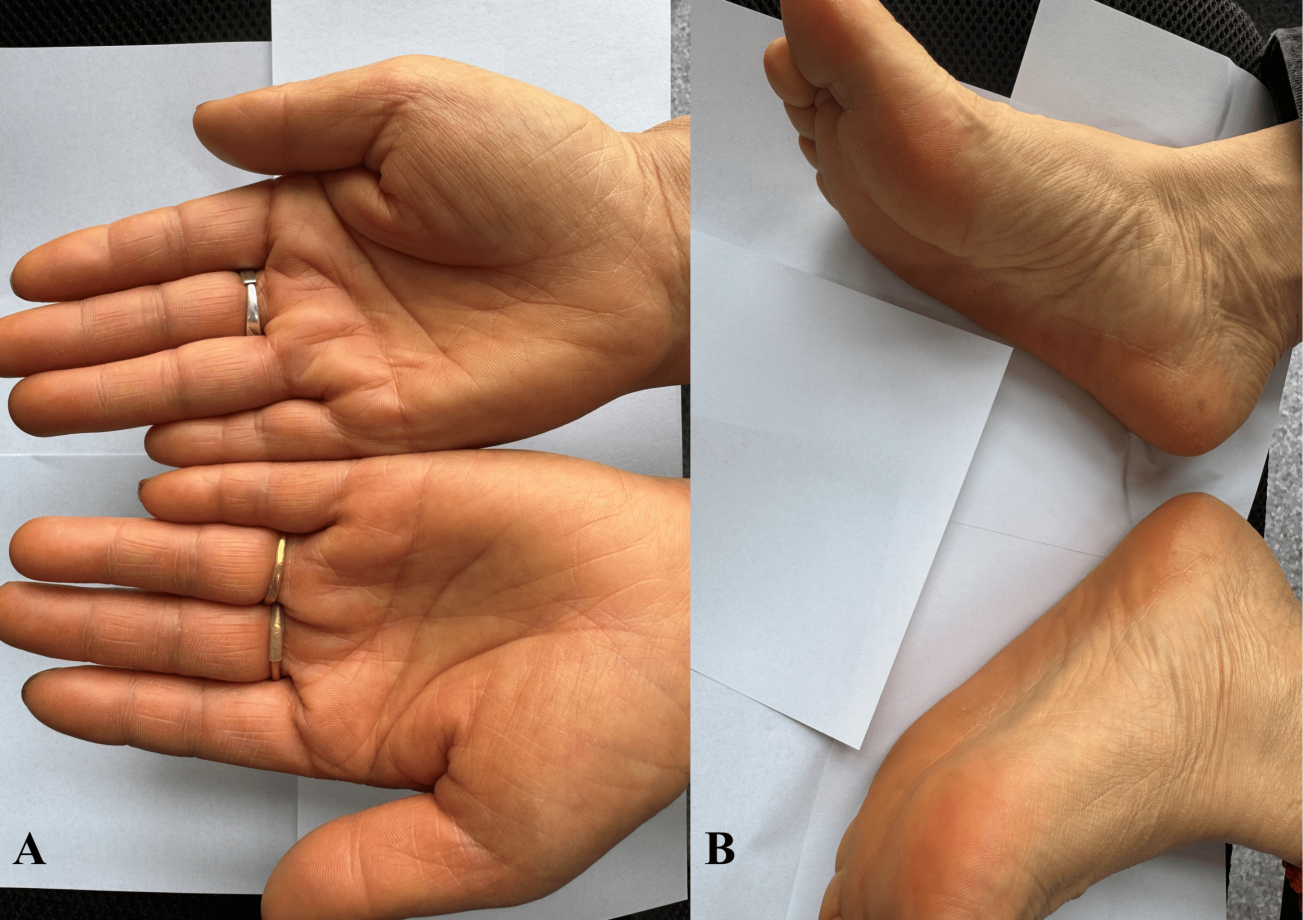
Patients typically present with orange-yellow skin pigmentation Yellow discoloration of the skin was observed on the patient's palms (A) and soles (B); however, no scleral icterus was present Patient privacy was protected in the images, and informed consent was obtained

The cutaneous manifestations of hypercarotenemia represent a fascinating example of how systemic metabolic alterations produce organ-specific clinical signs. The characteristic yellow-orange discoloration, termed carotenoderma, results from carotenoid deposition within the stratum corneum's lipid-rich intercellular matrix [33]. This predilection for keratinized epithelium explains the typical distribution pattern: most prominent on palms and soles (Figure 1), where the stratum corneum thickness ranges from 400 to 600 microns, significantly thicker than other parts of the body [34].

Dermoscopic examination reveals distinctive features differentiating carotenoderma from other causes of skin discoloration. Under polarized light, hypercarotenemic skin demonstrates a homogeneous yellow-orange hue without the greenish undertones characteristic of jaundice. Additionally, Wood's lamp examination may reveal orange fluorescence, particularly in severe cases, providing a non-invasive diagnostic aid [35].

The temporal dynamics of cutaneous changes provide diagnostic clues. Following the intake of carotenoids, the increase in carotenoid levels occurs relatively rapidly, typically within one to three days, whereas the renewal process of the stratum corneum is slower, taking two to three weeks [36]. This delay reflects the time required for epidermal turnover and carotenoid accumulation to visible levels. Conversely, following dietary restriction, improvement occurs gradually over 2-6 weeks, corresponding to epidermal renewal rates.

Laboratory Evaluation and Biomarkers

Contemporary laboratory assessment of hypercarotenemia has evolved beyond simple serum carotenoid quantification to encompass comprehensive metabolic profiling. High-performance liquid chromatography (HPLC) with photodiode array detection remains the gold standard for carotenoid speciation, differentiating β-carotene, α-carotene, lycopene, and other carotenoid species [37]. This differentiation has clinical relevance, as specific carotenoid patterns may suggest dietary sources or metabolic abnormalities.

Normal reference ranges require careful interpretation, considering dietary and demographic factors. While traditionally cited as 50-300 μg/dL for total carotenoids, population-based studies reveal significant variations based on dietary patterns, with vegetarians showing higher baseline levels [38]. More importantly, the ratio of different carotenoid species provides metabolic insights: elevated β-carotene with normal α-carotene suggests selective malabsorption or metabolic dysfunction rather than simple dietary excess [39].

Emerging biomarkers offer additional diagnostic refinement. The retinol:β-carotene ratio serves as a functional assessment of BCO1 activity. Additionally, measurement of retinol-binding protein 4 (RBP4) helps differentiate primary hypercarotenemia from vitamin A deficiency with compensatory carotenoid accumulation [40]. These functional assessments guide therapeutic decision-making beyond simple dietary counseling.

Imaging Modalities and Novel Diagnostic Approaches

Technological advances have introduced non-invasive methods for assessing tissue carotenoid status. Resonance Raman spectroscopy (RRS) enables real-time measurement of dermal carotenoid concentrations through detection of characteristic molecular vibrations [41]. Resonance Raman spectroscopy demonstrates a strong correlation with serum β-carotene levels, while reflection spectroscopy shows good agreement. These methods remain investigational and are not approved for routine clinical use.

Reflection spectroscopy represents another promising approach, utilizing specific wavelength absorption patterns to quantify cutaneous carotenoid content. Portable devices employing this technology enable point-of-care assessment, potentially facilitating early detection and treatment monitoring in primary care settings [42]. These technologies may prove particularly valuable in pediatric populations where venipuncture for serum testing presents challenges.

Advanced imaging modalities have revealed previously unrecognized manifestations of severe hypercarotenemia. Optical coherence tomography demonstrates increased dermal backscattering in affected individuals, while confocal microscopy reveals carotenoid deposits within keratinocyte cytoplasm and intercellular spaces [43]. These findings enhance understanding of the pathophysiology while potentially serving as severity markers in research settings.

Systematic diagnostic algorithm and clinical decision-making framework

**Figure 2:**
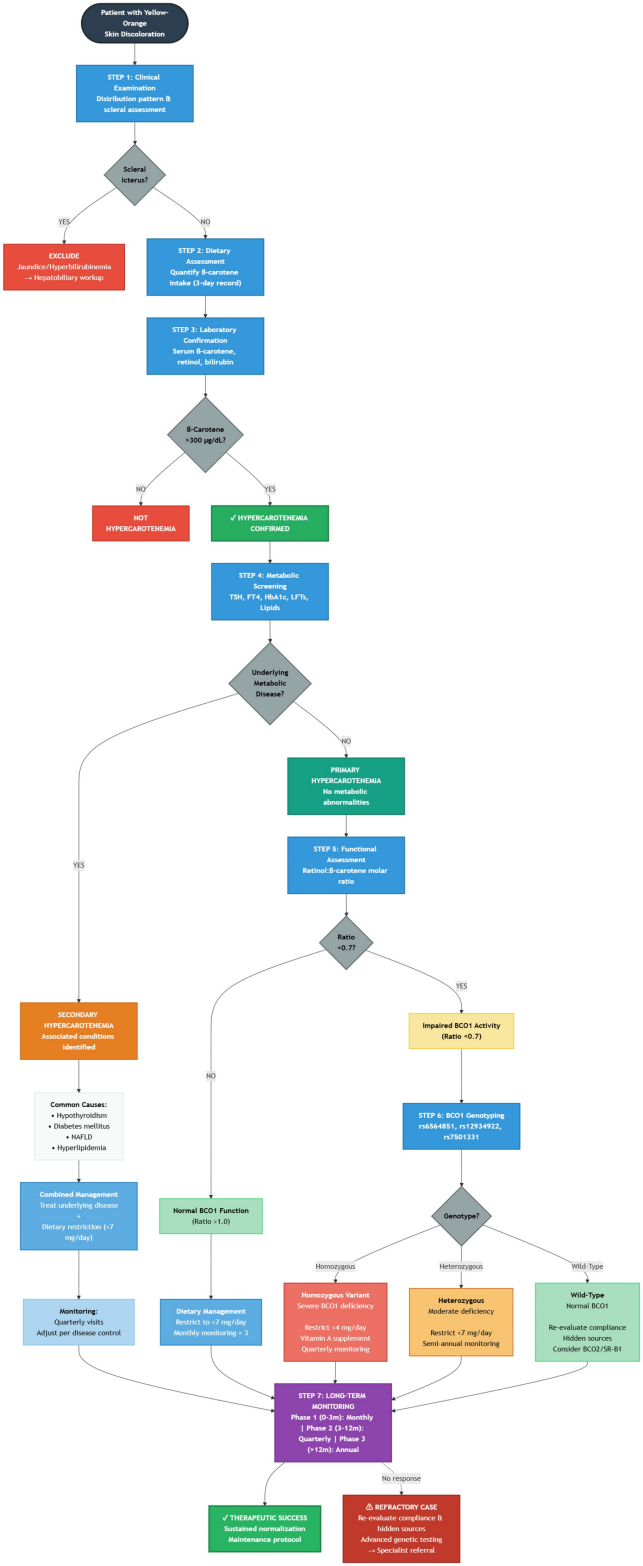
Comprehensive diagnostic algorithm for hypercarotenemia evaluation. Following initial clinical assessment and laboratory confirmation (Steps 1-3), the algorithm branches at Step 4 based on metabolic screening results. The left pathway addresses secondary hypercarotenemia associated with underlying conditions (hypothyroidism, diabetes, NAFLD, hyperlipidemia), while the right pathway evaluates primary hypercarotenemia through functional assessment (retinol:β-carotene molar ratio) and BCO1 genotyping when indicated. Both pathways converge at Step 7 for risk-stratified long-term monitoring. Decision points are represented by diamond shapes; management endpoints by rectangles. β-carotene diagnostic threshold: ≥300 μg/dL; genetic testing indicated when molar ratio <0.7 BCO1: beta-carotene oxygenase 1; TSH: thyroid-stimulating hormone; FT4: free thyroxine; HbA1c: hemoglobin A1c; LFTs: liver function tests; NAFLD: non-alcoholic fatty liver disease

The evaluation of hypercarotenemia requires a structured approach that balances diagnostic accuracy with clinical efficiency. We present a comprehensive seven-step algorithm (Figure 2) integrating clinical assessment, dietary evaluation, laboratory confirmation, metabolic screening, genetic testing, and personalized management. This framework facilitates evidence-based decision-making across diverse healthcare settings while identifying patients who may benefit from advanced genetic evaluation or specialist referral.

Step 1: Initial Clinical Evaluation and Differential Diagnosis

The diagnostic process begins with a systematic physical examination focusing on skin pigmentation distribution. Hypercarotenemia characteristically presents with yellow-orange discoloration affecting palms, soles, nasolabial folds, and forehead - areas with thick stratum corneum. The critical distinguishing feature is complete sparing of the sclerae, which differentiates hypercarotenemia from jaundice and hyperbilirubinemia. If scleral icterus is present, the evaluation must immediately divert to hepatobiliary workup, including serum bilirubin and comprehensive liver function tests. Scleral sparing remains the most reliable clinical sign to proceed with hypercarotenemia assessment.

Step 2: Comprehensive Dietary Assessment

Once hypercarotenemia is clinically suspected, a structured dietary history becomes essential. A three-day food record should quantify β-carotene intake from major sources: carrots (10-12 mg/100g), sweet potatoes (6-8 mg/100g), spinach (8-10 mg/100g), carrot juice (20-30 mg/glass), and supplements (15-50 mg/dose). Patients can be risk-stratified by daily intake: low risk (<10 mg/day), moderate risk (10-20 mg/day), high risk (>20 mg/day), and very high risk (>30 mg/day). Individuals developing hypercarotenemia with intake <10 mg/day should be prioritized for genetic evaluation, suggesting significant BCO1 enzyme deficiency. Conversely, extremely high dietary consumption (>30 mg/day) may produce hypercarotenemia even in those with normal BCO1 activity.

Step 3: Laboratory Confirmation

Laboratory testing provides definitive diagnosis and severity quantification. The core panel includes: (1) serum β-carotene by high-performance liquid chromatography (diagnostic threshold ≥300 μg/dL; severe elevation >500 μg/dL), (2) serum retinol to assess conversion efficiency and vitamin A status (normal 30-70 μg/dL), and (3) total bilirubin to confirm absence of hyperbilirubinemia (must be <1.2 mg/dL). A serum β-carotene concentration ≥300 μg/dL with normal bilirubin confirms a hypercarotenemia diagnosis. Levels exceeding 500 μg/dL indicate severe accumulation, suggesting either very high dietary intake or significant metabolic impairment requiring intensive intervention.

Step 4: Screening for Secondary Causes

Before attributing hypercarotenemia solely to dietary excess, underlying metabolic conditions must be systematically excluded. The recommended panel includes: thyroid function tests (TSH, free T4)-hypothyroidism reduces BCO1 expression; glucose metabolism assessment (fasting glucose or HbA1c)-diabetes affects carotenoid transport; hepatic function tests (ALT, AST)-liver disease impairs clearance; and lipid profile-dyslipidemia alters lipoprotein-mediated carotenoid distribution. Based on results, cases are classified as primary hypercarotenemia (dietary excess without metabolic abnormalities) or secondary hypercarotenemia (associated with identifiable conditions). Secondary cases require concurrent treatment of the underlying disorder alongside dietary modification for optimal outcomes.

Step 5: Functional Assessment and Genetic Testing Indications

For confirmed primary hypercarotenemia, the retinol:β-carotene molar ratio assesses BCO1 functional capacity and guides genetic testing decisions. Calculate as: serum retinol (μmol/L) ÷ serum β-carotene (μmol/L). Interpretation: ratio ≥1.0 indicates normal BCO1 activity (dietary excess likely); ratio 0.7-1.0 suggests borderline efficiency (consider heterozygous variant); ratio <0.7 indicates significant impairment (genetic testing strongly recommended). Additional indications for BCO1 genotyping include: refractory hypercarotenemia despite strict dietary restriction (<7 mg/day for three months), development at low intake (<15 mg/day), positive family history, recurrent episodes, young age at presentation (<30 years), or severe elevation (>600 μg/dL) with modest intake.

Step 6: BCO1 Genotyping and Interpretation

Targeted single-nucleotide polymorphism analysis identifies variants associated with reduced enzyme activity: rs6564851 (T/G), rs12934922 (A/G), and rs7501331 (T/C). Management is genotype-dependent. Homozygous variant carriers exhibit severe BCO1 deficiency (50-70% activity reduction) requiring strict β-carotene restriction (<4 mg/day), preformed vitamin A supplementation (3,000-5,000 IU daily), and quarterly monitoring. Heterozygous carriers show moderate deficiency (25-35% reduction) managed with moderate restriction (<7-10 mg/day) and semi-annual monitoring. Wild-type individuals with normal BCO1 function require re-evaluation of dietary compliance, identification of hidden sources, and consideration of alternative genetic factors (BCO2, SR-B1, APOE variants).

Step 7: Risk-Stratified Monitoring Protocol

Systematic follow-up ensures therapeutic success while preventing vitamin A deficiency. Phase 1 (Active Correction, zero to three months): monthly visits with serum carotenoids at months one and three; target β-carotene <300 μg/dL. Phase 2 (Stabilization, 3-12 months): quarterly assessments with serum carotenoids and retinol every three months; establish a sustainable dietary threshold maintaining levels 150-250 μg/dL. Phase 3 (Maintenance, >12 months): annual comprehensive review with yearly carotenoid and vitamin A assessment; periodic metabolic re-screening every two to three years. Refractory cases failing standard intervention after six months require systematic re-evaluation, including compliance verification, identification of hidden sources, advanced genetic testing, and potential specialist referral to metabolic or nutrition centers.

Clinical Implementation

This algorithm provides a practical roadmap applicable from primary care to specialized metabolic clinics. Primary care physicians can manage initial recognition, dietary counseling, and straightforward cases, while complex presentations with refractory symptoms, genetic variants, or multiple comorbidities benefit from specialist collaboration. Emerging technologies, including point-of-care Raman spectroscopy, rapid genetic screening panels, and artificial intelligence-driven dietary tracking, promise to enhance diagnostic efficiency. By implementing this structured approach, clinicians can optimize diagnostic accuracy and therapeutic effectiveness while personalizing management based on individual genetic and metabolic profiles.

Evidence-based management strategies

Structured Dietary Interventions

The cornerstone of hypercarotenemia management remains dietary modification, though implementation requires sophistication beyond simple carotenoid restriction. Effective intervention necessitates a comprehensive nutritional assessment to prevent inadvertent micronutrient deficiencies while addressing carotenoid excess. A structured approach involves quantitative dietary analysis using validated food frequency questionnaires specifically designed to capture carotenoid intake, followed by personalized meal planning, maintaining nutritional adequacy [44].

Clinical trials and toxicity studies have shown that β-carotene supplements are generally safe at doses ranging from 15-50 mg/day, though hypercarotenemia can develop at these levels in susceptible individuals [45]. Therapeutic intervention typically involves eliminating concentrated sources (carrot juice, sweet potato, pumpkin) while maintaining moderate intake of green leafy vegetables, providing essential folate, vitamin K, and minerals. Importantly, complete carotenoid elimination is neither necessary nor advisable given their antioxidant properties and role as vitamin A precursors [45].

The reintroduction phase requires careful monitoring to establish individual tolerance thresholds. Serial measurements of serum carotenoids during graduated dietary challenges help identify personal limits, typically ranging from 2-4.8 mg daily β-carotene in metabolically normal individuals [46]. This personalized approach prevents recurrence while maximizing the health benefits of carotenoid-rich foods.

Therapeutic Considerations in Special Populations

Management complexity increases substantially in populations with underlying metabolic conditions. Hypothyroid patients require concurrent thyroid hormone optimization, with studies demonstrating that achieving euthyroid status can reduce serum carotenoids independent of dietary changes [47, 48]. This highlights the importance of addressing underlying metabolic dysfunction rather than focusing solely on dietary restriction.

Diabetic patients present unique challenges given the potential benefits of carotenoids for glycemic control and diabetic complications. Rather than strict limitation, management focuses on optimizing carotenoid species consumed-emphasizing lycopene and lutein over β-carotene-while improving overall metabolic control. Continuous glucose monitoring data suggest that carotenoid-rich meals within a low-glycemic index dietary pattern may actually improve postprandial glucose excursions [49].

The approach to hypercarotenemia in eating disorders requires particular sensitivity. While normalization of carotenoid levels serves as one marker of recovery, aggressive dietary manipulation may exacerbate food-related anxiety. Management integrates with comprehensive eating disorder treatment, with carotenoid monitoring serving as an objective marker of nutritional rehabilitation and adipose tissue restoration [6].

Monitoring Protocols and Risk Stratification

Effective management extends beyond acute intervention to encompass systematic monitoring, preventing recurrence while optimizing health outcomes. Evidence-based protocols suggest initial monthly assessments during the correction phase, including clinical evaluation of skin discoloration and serum carotenoid quantification. Following normalization, quarterly monitoring for 12 months establishes stability before transitioning to annual surveillance.

Comprehensive monitoring encompasses more than carotenoid levels alone. Regular assessment includes vitamin A status (serum retinol, RBP4), thyroid function in at-risk populations, and markers of oxidative stress to ensure adequate antioxidant status despite carotenoid restriction. This holistic approach identifies individuals requiring supplementation with alternative antioxidants or preformed vitamin A [50].

Systems Biology Approaches to Carotenoid Metabolism

The application of systems biology methodologies has revolutionized the understanding of carotenoid metabolism complexity. Integrated multi-omics analyses combining genomics, transcriptomics, proteomics, and metabolomics reveal previously unrecognized metabolic networks identifying carotenoid metabolism as a hub connecting lipid metabolism, circadian rhythms, immune function, and cellular stress responses [51].

Metabolic flux analysis using stable isotope-labeled carotenoids has quantified pathway dynamics, revealing tissue-specific differences in carotenoid processing. Adipose tissue emerges not merely as a storage depot but as an active metabolic site, with adipocyte BCO1 expression responding to metabolic status and potentially contributing to systemic carotenoid homeostasis. These findings suggest that adiposity and body composition may influence hypercarotenemia susceptibility beyond simple storage capacity [52].

The gut-liver axis represents another frontier in carotenoid metabolism research. Intestinal organoid models demonstrate that enterocyte carotenoid metabolism exhibits circadian variation, with peak BCO1 expression during active feeding periods. Additionally, bile acid composition influences carotenoid absorption efficiency, while hepatic FXR/RXR signaling integrates carotenoid and cholesterol metabolism [53].

Emerging research frontiers

The convergence of genetic understanding, metabolic profiling, and digital health technologies enables unprecedented personalization in hypercarotenemia prevention and management. Polygenic risk scores incorporating variants across multiple carotenoid metabolism genes show promise for identifying high-risk individuals before clinical manifestations. When combined with dietary assessment apps utilizing image recognition and artificial intelligence, real-time monitoring and intervention become feasible [54].

Novel therapeutic approaches under investigation extend beyond dietary modification. Small molecule enhancers of BCO1 activity, identified through high-throughput screening, show promise in preclinical models for accelerating carotenoid clearance. Additionally, engineered probiotics expressing carotenoid-degrading enzymes may offer a biological approach to reducing absorption in high-risk individuals [55].

The development of carotenoid analogs with modified metabolism represents another innovative approach. Synthetic carotenoids designed for enhanced water solubility and rapid clearance could provide antioxidant benefits without accumulation risk. Early-stage compounds show promising profiles in animal models, though human studies remain years away. These developments may ultimately enable harnessing carotenoid benefits while minimizing hypercarotenemia risk in susceptible populations [56].

Future research priorities should focus on: (1) randomized controlled trials investigating ISX transcription factor modulation as a therapeutic target, (2) microbiome engineering approaches using carotenoid-metabolizing probiotics, (3) genotype-guided nutrition interventions with standardized protocols, and (4) development of point-of-care genetic testing for BCO1 variants to enable personalized dietary counseling.

## Conclusions

Hypercarotenemia is a visible yet metabolically informative condition where dietary exposure, genetic susceptibility, and systemic dysfunction intersect. Current evidence clarifies a tightly regulated network centered on BCO1/BCO2 enzymes and ISX-mediated feedback, with common BCO1 variants (such as rs12934922/rs7501331) reducing enzymatic activity and amplifying interindividual variability in carotenoid handling. Clinically, the characteristic palmoplantar yellow-orange discoloration with scleral sparing is pivotal for distinguishing it from jaundice, preventing unnecessary hepatobiliary imaging or invasive procedures - an issue frequently encountered in surgical practice. Modern diagnostics (HPLC carotenoid profiling, retinol:β-carotene ratio, and emerging Raman spectroscopy) support precise phenotyping. The epidemiologic context-wider adoption of plant-forward diets and rising hypothyroidism, diabetes, and fatty liver-has expanded the at‑risk population, while eating disorders highlight paradoxical elevations independent of intake.

Management has shifted from simple restriction to precision nutrition: aligning carotenoid intake with genotype, metabolic status, and comorbidities, while preserving the antioxidant and immune benefits of carotenoids. Addressing primary disorders (thyroid optimization, glycemic control, NAFLD care) often normalizes carotenoid levels as effectively as dietary change, and variant carriers may require stricter β‑carotene limits. Looking ahead, translating mechanistic insights into clinical tools should be prioritized: pragmatic BCO1 genotyping for risk stratification, validated prediction models integrating metabolic markers, and trials targeting ISX/BCO1 pathways. From a hepatobiliary perspective, recognizing hypercarotenemia as a biomarker of systemic metabolism - not merely a cosmetic issue - can streamline care, reduce costs, and prompt early intervention for associated conditions. Integrating nutrigenomics with clinical practice will enable individualized recommendations that maximize the health benefits of carotenoid-rich diets while preventing excess accumulation in genetically and metabolically susceptible patients.
